# Comprehensive Insights into Natural Bioactive Compounds: From Chemical Diversity and Mechanisms to Biotechnological Innovations and Applications

**DOI:** 10.1002/open.202500469

**Published:** 2025-11-17

**Authors:** Sajid Ali, Atif Ali Khan Khalil, Muhammad Saeed Akhtar, Adnan Amin, Wajid Zaman

**Affiliations:** ^1^ Department of Horticulture and Life Science Yeungnam University Gyeongsan Republic of Korea; ^2^ Department of Biotechnology Yeungnam University Gyeongsan Republic of Korea; ^3^ Department of Chemistry Yeungnam University Gyeongsan Republic of Korea; ^4^ Department of Life Sciences Yeungnam University Gyeongsan Republic of Korea

**Keywords:** antioxidant mechanisms, biotechnological production, chemical diversity, metabolomics, natural bioactive compounds, pharmacokinetics

## Abstract

Natural bioactive compounds derived from plants, microbes, and marine organisms represent a rich and diverse reservoir of structurally complex molecules with a broad spectrum of biological activities. This review comprehensively explores the chemical diversity of these compounds, spanning major classes such as alkaloids, flavonoids, terpenoids, phenolics, and glycosides, and elucidates the molecular mechanisms underlying antioxidant, anti‐inflammatory, antimicrobial, anticancer, neuroprotective, and cardiovascular effects. A novel contribution of this review is its emphasis on the integration of advanced technologies that are reshaping natural product research. Biotechnological approaches, including plant cell culture, microbial fermentation, and metabolic engineering, support more sustainable and scalable production. Nanotechnology‐based delivery systems enhance bioavailability and therapeutic performance by addressing pharmacokinetic challenges. Artificial intelligence enables faster screening, structural analysis, and activity prediction, significantly accelerating discovery and development. These interdisciplinary strategies also help overcome challenges such as low yield, toxicity, chemical variability, and environmental concerns. The review further discusses diverse industrial applications in pharmaceuticals, agriculture, food, cosmetics, and nutraceuticals. By highlighting the combined use of biotechnology, nanotechnology, and AI‐driven tools, this review underscores a new paradigm in the sustainable and efficient utilization of natural bioactive compounds for both health and industry.

## Graphical abstract

Natural products derived from plants, animals, fungi, bacteria, and minerals contain diverse bioactive classes such as alkaloids, flavonoids, terpenoids, saponins, tannins, and phenolics. These natural products work through different mechanisms, including ROS inhibition, NF‐κB suppression, and cytokine regulation, and exhibit wide applications across pharmaceutical, cosmetic, agricultural, and food industries.

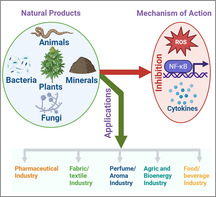

## 1 Introduction

Natural bioactive compounds, predominantly sourced from plants but also derived from microbes and marine organisms, have played a pivotal role in the development of therapeutic agents, functional foods, and industrial products for centuries [1, 2]. The rich history of natural products is deeply intertwined with traditional medicine systems worldwide, such as Ayurveda in India, traditional Chinese medicine, and various indigenous healing practices [3, 4]. These systems have utilized plant extracts and isolated compounds to treat a myriad of diseases long before the advent of modern pharmaceuticals. For example, morphine, isolated from the opium poppy (*Papaver somniferum*), revolutionized pain management, while quinine, extracted from the bark of *Cinchona* species, has been instrumental in combating malaria [5, 6]. Thus, the foundations for contemporary drug discovery and development were provided by these natural products, thereby highlighting the enduring relevance of natural compounds as pharmacological templates. Meanwhile, the profound biochemical complexity and structural diversity of these molecules offer unique advantages, including highly specific interactions with biological targets and relatively fewer side effects compared to many synthetic chemicals [7].

Over recent decades, multiple converging factors have fueled a marked resurgence in interest in natural bioactive compounds. However, growing concerns regarding the safety profiles, environmental impacts, and the rising incidence of resistance associated with synthetic drugs and agrochemicals have catalyzed a global shift toward exploring safer, more sustainable alternatives derived from natural sources [8]. The World Health Organization estimates that the primary healthcare needs of approximately 80% of the global population depend on herbal medicines and natural products, underscoring the vital role of these compounds in global health [9]. Moreover, advances in technologies such as high‐performance liquid chromatography (HPLC), nuclear magnetic resonance (NMR) spectroscopy, and mass spectrometry (MS) have enabled the identification, structural elucidation, and quantitative analysis of an immense variety of natural compounds [10]. Coupled with progress in molecular biology and bioinformatics, these tools have unveiled intricate molecular interactions and mechanisms of action, providing scientific validation for traditional uses and revealing new therapeutic potentials [11]. Recent developments in artificial intelligence (AI) and machine learning (ML) have further supported rapid prediction of natural compound bioactivity, target interactions, and pharmacokinetics, thereby reducing reliance on trial‐and‐error‐based screening and accelerating early‐stage discovery [12, 13, 14]. These data‐driven tools complement experimental advances and help uncover novel structure–activity relationships that guide rational natural product design.

The chemical diversity of natural bioactive compounds is vast and encompasses multiple major classes, including alkaloids, flavonoids, terpenoids, phenolics, glycosides, saponins, and polysaccharides [15]. The physicochemical properties and biological activities are governed by distinct structural motifs exhibited by each class. Alkaloids, characterized by nitrogen‐containing heterocycles, often exhibit potent pharmacological activities, including analgesic, antimalarial, and anticancer effects [16]. Flavonoids, a large family of polyphenolic compounds, are well‐recognized for possessing antioxidant, anti‐inflammatory, and cardioprotective properties through modulating signaling pathways and enzyme inhibition [17, 18]. Terpenoids, the largest group of natural products, encompass molecules with diverse roles ranging from antimicrobial to anticancer activity, exemplified by artemisinin, a sesquiterpene lactone used to treat malaria [19]. These compounds exert biological effects through complex mechanisms, including scavenging reactive oxygen species (ROS), modulating transcription factors such as nuclear factor kappa‐light‐chain‐enhancer of activated B cells (NF‐κB), and regulating enzymes such as cyclooxygenase (COX) [20]. Nevertheless, the interplay between chemical structure and biological function remains a major area of research, driving the discovery of novel compounds with enhanced efficacy and specificity.

Beyond their intrinsic biological importance, natural bioactive compounds occupy key roles across various industrial sectors. Notably, the pharmaceutical industry has harnessed these compounds to develop a wide range of approved drugs and promising drug leads, addressing diseases ranging from cancer and infectious diseases to metabolic and neurodegenerative disorders [21]. Agricultural applications include the use of natural pesticides, herbicides, and biostimulants, which reduce reliance on synthetic chemicals, thereby promoting sustainability and environmental safety [22, 23]. Meanwhile, in the food industry, natural antioxidants and preservatives derived from bioactive compounds contribute to extending shelf life and enhancing nutritional quality [24]. The cosmetic and nutraceutical industries also utilize these compounds for their antiaging, photoprotective, and health‐promoting properties, responding to increasing consumer demand for “clean‐label” and natural ingredients [25, 26]. This diverse spectrum of applications highlights the need for a comprehensive understanding of not only the chemical and biological characteristics of these compounds but also their production, formulation, safety, and regulatory compliance. Yet, there is currently a lack of integrative reviews that explore how recent advances in biotechnology, artificial intelligence, and nanotechnology collectively enhance the scalable, safe, and sustainable development of these compounds. This knowledge gap limits strategic progress across both research and industrial pipelines. Recent developments in microbial fermentation and synthetic biology have demonstrated the potential for scalable and environmentally responsible production of natural compounds [27, 28]. Artificial intelligence, including deep learning and graph‐based approaches, is enabling rapid prioritization, predictive modeling, and target prediction for natural products [29, 30]. At the same time, nanotechnology has facilitated improved delivery systems and pharmacokinetic profiles for natural bioactives, helping to overcome longstanding barriers in solubility and stability [31, 32].

The advent of modern biotechnology has markedly enhanced the production, characterization, and application of natural bioactive compounds [33]. Plant cell cultures, microbial fermentation, and metabolic engineering enable the sustainable and scalable production of valuable molecules, circumventing limitations related to natural resource depletion and environmental variability [34]. Genetic engineering and synthetic biological approaches can facilitate the biosynthesis of novel derivatives and improved yields. Analytical advances, including metabolomics and high‐throughput screening, can accelerate the identification and functional characterization of bioactive molecules [35]. Furthermore, nanotechnology‐driven delivery systems improve the solubility, stability, and targeted delivery of natural compounds, enhancing therapeutic efficacy while reducing toxicity [36]. Regulatory frameworks and safety evaluations have become increasingly stringent, ensuring the quality, efficacy, and protection of products and consumers. Standardization and authentication techniques, such as DNA barcoding and chromatographic fingerprinting, guarantee reproducibility and traceability in natural product‐based formulations [37].

This review comprehensively explores current research on natural bioactive compounds by integrating multiple dimensions: the chemical diversity and classification of these compounds, detailed mechanisms of biological action, and broad‐spectrum applications in health, agriculture, and industry. Moreover, this review thoroughly evaluates advances in biotechnological production methods and analytical technologies, alongside an in‐depth assessment of safety, toxicity, and regulatory issues. Most importantly, it presents a novel and holistic synthesis of how artificial intelligence, biotechnological engineering, and nanodelivery systems converge to address longstanding challenges in bioactive compound development. Indeed, by presenting a holistic perspective, this work aims to bridge the gaps between fundamental research and practical application, supporting ongoing innovation and the responsible utilization of natural bioactive compounds.

## 2 Diversity of Natural Bioactive Components

Natural bioactive compounds exhibit remarkable structural and functional diversity, reflecting the evolutionary adaptation of organisms to their ecological niches. These compounds span a wide chemical spectrum, each class distinguished by unique molecular frameworks that determine their physicochemical properties and biological functions [38]. Furthermore, the diversity of these compounds underpins a broad range of pharmacological activities and industrial applications. Thus, understanding this chemical heterogeneity is essential for the targeted discovery, extraction, and application of bioactives, as the molecular architecture of each compound dictates its interaction with biological targets and therapeutic potential. This section provides an overview of the major chemical classes of natural bioactive compounds, elaborating on their characteristic structures and roles, followed by a discussion of their natural sources and extraction methodologies.

### 2.1 Classification of Bioactive Compounds

Natural bioactive compounds are traditionally classified into several major chemical families, each characterized by distinct structural motifs and biological activities. Among these, alkaloids represent a prominent group of nitrogen‐containing heterocyclic compounds with potent pharmacological effects. Alkaloids such as morphine, extracted from *Papaver somniferum*, and quinine, derived from *Cinchona* bark, have long been valued for their analgesic and antimalarial properties, respectively [5, 6]. The presence of nitrogen atoms within the alkaloid structure facilitates interactions with neurotransmitter receptors and enzymes, conferring notable biological specificity [39]. These compounds often exhibit complex ring systems and stereochemistry critical for their bioactivity.

Another diverse group, flavonoids, comprises polyphenolic compounds renowned for their antioxidant, anti‐inflammatory, and cardioprotective effects. Examples include quercetin and kaempferol, which are widely distributed in fruits and vegetables [17, 40]. Flavonoids typically feature two aromatic rings connected by a three‐carbon bridge, forming a variety of subclasses, including flavones, flavonols, and flavanones. The free radical scavenging activity of flavonoids comes from their ability to donate hydrogen atoms and chelate metal ions [41]. Additionally, flavonoids modulate cellular signaling pathways, including those involving NF‐κB and MAPKs, which play pivotal roles in inflammation and cancer [40, 42].

Terpenoids, also known as isoprenoids, constitute the largest and most structurally diverse class of natural products [43]. Derived biosynthetically from isoprene units, terpenoids range from simple monoterpenes to complex diterpenes and triterpenes. Notably, artemisinin, a sesquiterpene lactone from *Artemisia annua*, has demonstrated exceptional antimalarial and anticancer properties [44]. Terpenoids interact with multiple biological targets, including ion channels, enzymes, and cell membranes, contributing to their broad‐spectrum bioactivity [45]. Other important classes include phenolics, which encompass simple phenols to complex tannins with antioxidant and antimicrobial properties; glycosides, compounds where sugars are bound to aglycones, influencing solubility and activity; saponins, known for their surfactant properties and immunomodulatory effects [46]. The specific functional groups present in these compounds, such as hydroxyl, carbonyl, and amine, significantly influence their bioavailability, metabolism, and mechanism of action. This highlights the importance of detailed structural elucidation in drug development and industrial applications. Figure 1 presents the representative chemical structures of these compound classes to illustrate their core features and functional groups critical for activity.

FIGURE 1Chemical structures of representative bioactive alkaloids, flavonoids, and terpenoids reported from natural sources.

**Figure:**
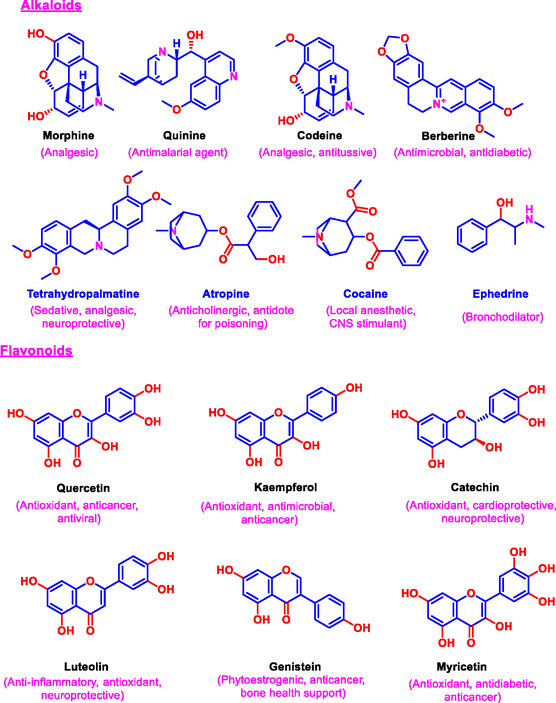

**Figure:**
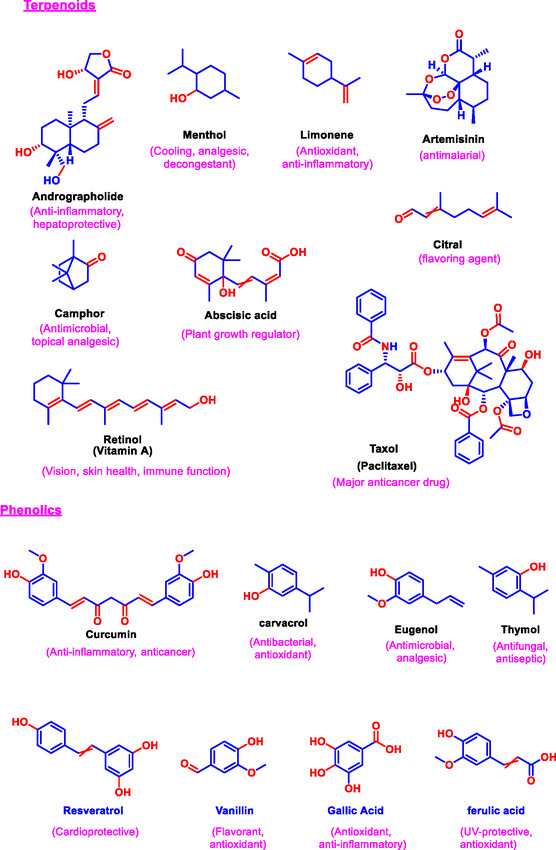

**Figure:**
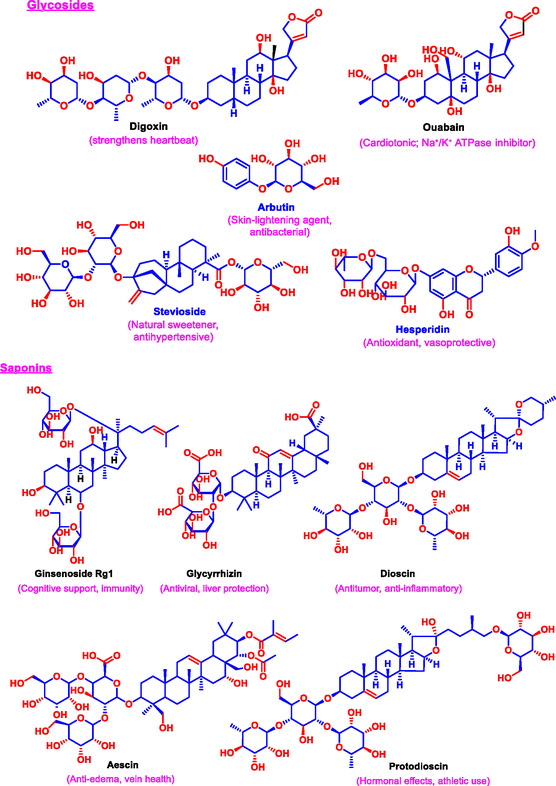

### 2.2 Sources and Extraction Methods

The sourcing of natural bioactive compounds encompasses a diverse range of biological origins, primarily from medicinal herbs, cultivated crops, wild plants, and, increasingly, endophytic microorganisms residing within plant tissues. Medicinal plants such as *Hypericum perforatum* (St. John's Wort), *Ginkgo biloba*, and *Curcuma longa* have historically served as rich reservoirs of pharmacologically active compounds [47]. Additionally, agro‐industrial by‐products and marine organisms are emerging as sustainable sources [48]. The choice of source material directly impacts the yield and spectrum of bioactive molecules, as environmental factors, plant maturity, and genetic variability influence secondary metabolite profiles.

Extraction techniques have evolved to optimize the isolation of these compounds, balancing efficiency, selectivity, and environmental considerations [49]. Traditional solvent extraction, employing polar (e.g., methanol, ethanol) or nonpolar solvents (e.g., hexane), remains widely used due to its simplicity and scalability. However, conventional methods often suffer from drawbacks such as lengthy extraction times, solvent toxicity, and low selectivity [50]. Subsequent advances in extraction technologies have introduced greener and more efficient approaches: supercritical fluid extraction (SFE), notably using carbon dioxide (CO_2_), enables selective, solvent‐free extraction with minimal thermal degradation; ultrasound‐assisted extraction (UAE) utilizes cavitation to disrupt plant matrices, enhancing solvent penetration and extraction rates; microwave‐assisted extraction (MAE) employs microwave energy to heat intracellular water, accelerating compound release [51, 52, 53]. These innovative methods reduce solvent use, lower energy consumption, and improve yields and compound stability.

Therefore, the choice of extraction method influences not only the quantity but also the quality and bioactivity of the isolated compounds. Furthermore, factors such as solvent polarity, temperature, pressure, and time govern the selectivity toward specific chemical classes. For example, polar solvents favor the extraction of flavonoids and phenolics, while nonpolar solvents are preferred for terpenoids [54, 55]. Additionally, extraction conditions must be optimized to prevent degradation of thermolabile compounds and preserve bioactivity. The increasing demand for sustainable and food‐grade extracts propels the adoption of eco‐friendly extraction processes. Table 1 presents a comparative summary of common extraction techniques, including solvent types, extraction times, yields, selectivity, advantages, and limitations, to facilitate method selection.

**TABLE 1:** Comparative summary of extraction methods for natural bioactive compounds.

Source/method	Solvent type	Extraction time	Selectivity	Advantages	Limitations	References
Medicinal plants (e.g., *Curcuma longa*)	Polar (ethanol, methanol), nonpolar (hexane)	Hours to days	Flavonoids, phenolics (polar); terpenoids, alkaloids (nonpolar)	Simple, scalable, widely used	Long time, solvent toxicity, low selectivity	[56, 57]
SFE	Supercritical CO_2_ + cosolvents (ethanol)	30 min to 2 h	Nonpolar compounds, some polar with co‐solvents	Solvent‐free, selective, preserves thermolabile compounds	Expensive equipment, method optimization needed	[58, 59]
UAE	Polar/nonpolar solvents	Minutes to 1 h	Phenolics, flavonoids, alkaloids	Faster extraction, low solvent use	Scale‐up challenges, potential cavitation damage	[60, 61]
MAE	Mostly polar solvents	5–30 min	Polar compounds like phenolics, flavonoids	Rapid, energy‐efficient, improved yield	Initial cost, possible thermal degradation	[62, 63, 64]
Conventional solvent extraction	Polar/nonpolar solvents	Hours to days	Broad spectrum depending on solvent polarity	Simple, widely applicable	Time‐consuming, environmental concerns	[65, 66, 67]
Enzyme‐assisted extraction (EAE)	Water or buffer systems	Several hours	Polysaccharides, phenolics	Mild conditions, enhanced yield and purity	Enzyme cost, longer process	[68, 69, 70]
Pressurized liquid extraction (PLE)/accelerated solvent extraction (ASE)	Polar/nonpolar solvents	Minutes to 1 h	Wide range, including phenolics, terpenoids	Fast, automated, reduced solvent consumption	Equipment cost, optimization needed	[71, 72, 73]
Microwave‐assisted hydrodistillation (MAHD)	Water (steam)	Minutes to 1 h	Volatile oils, essential oils	Faster than traditional distillation, energy saving	Equipment complexity	[74, 75, 76]
Ionic liquid‐based extraction	Ionic liquids	Variable	Wide chemical range, tunable selectivity	Green solvents, recyclable, highly selective	High cost, toxicity concerns for some ILs	[77, 78]
Deep eutectic solvent extraction (DES)	Deep eutectic solvents	Minutes to hours	Phenolics, flavonoids, alkaloids	Eco‐friendly, biodegradable, low toxicity	Emerging tech, scale‐up challenges	[79, 80, 81]

## 3 Biological Activities and Mechanisms of Action

Natural bioactive compounds exhibit a broad spectrum of biological activities that underpin their therapeutic potential and diverse applications [38]. These activities arise from complex interactions at the molecular and cellular levels, modulating key physiological pathways that are involved in health and disease. The biological effects include antioxidant, anti‐inflammatory, antimicrobial, anticancer, neuroprotective, and cardiovascular actions, among others [82, 83]. Hence, understanding these mechanisms is crucial for the rational development of natural product‐based therapeutics and functional ingredients. This section elaborates on the principal biological activities of natural bioactives and their underlying molecular mechanisms.

### 3.1 Antioxidant and Anti‐Inflammatory Activities

The antioxidant activity of natural compounds is one of the most extensively studied biological functions. Oxidative stress, caused by the accumulation of ROS and free radicals, is implicated in the pathogenesis of numerous chronic diseases, including cancer, cardiovascular disorders, and neurodegeneration [84]. Natural antioxidants mitigate oxidative damage by directly scavenging ROS, chelating transition metal ions that catalyze ROS formation, and upregulating endogenous antioxidant defense enzymes such as superoxide dismutase (SOD), catalase, and glutathione peroxidase. These multipronged antioxidant effects preserve cellular integrity and function [85].

**FIGURE 2:**
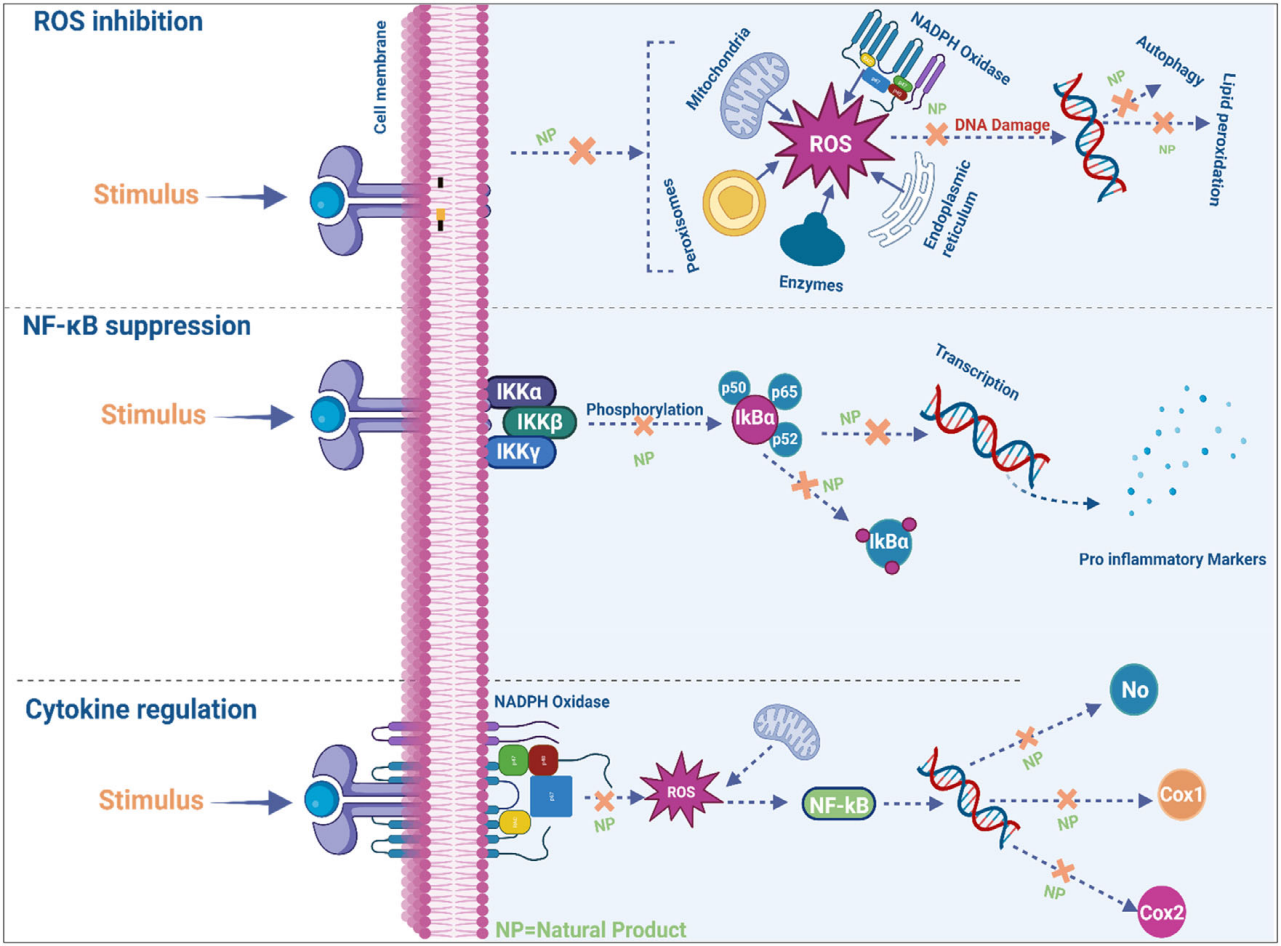
Signaling pathways modulated by natural products, including ROS neutralization and NF‐κB inhibition.

Closely linked to antioxidant action, the anti‐inflammatory effects of natural compounds are achieved through the modulation of inflammatory signaling pathways [86]. Chronic inflammation is a driver of many pathological conditions and is characterized by elevated proinflammatory cytokines, such as tumor necrosis factor‐alpha (TNF‐α) and interleukin‐6 (IL‐6) [87]. Natural bioactives suppress these cytokines by inhibiting key transcription factors, such as NF‐κB and signal transducers and activators of transcription (STATs) [88]. Additionally, they inhibit enzymes such as cyclooxygenase‐2 (COX‐2) and modulate mitogen‐activated protein kinase (MAPK) pathways, reducing the synthesis of inflammatory mediators [89]. Classic examples include curcumin from *Curcuma longa* and resveratrol from grapes, which have demonstrated strong antioxidant and anti‐inflammatory properties in vitro and in vivo. Additionally, they inhibit enzymes such as cyclooxygenase‐2 (COX‐2) and modulate mitogen‐activated protein kinase (MAPK) pathways, reducing the synthesis of inflammatory mediators [89] (Figure 2).

### 3.2 Antimicrobial and Antiviral Activities

Natural bioactive compounds also exhibit broad‐spectrum antimicrobial activity against Gram‐positive and Gram‐negative bacteria, fungi, and viruses [90]. The mechanisms through which natural bioactive compounds function include disrupting microbial cell membranes, inhibiting nucleic acid and protein synthesis, and interfering with microbial energy metabolism [91]. For instance, phenolic compounds can permeabilize bacterial membranes, leading to leakage of cellular contents and death [92]. Meanwhile, flavonoids and terpenoids have been shown to inhibit DNA gyrase and reverse transcriptase enzymes, which are critical for microbial replication [93].

Emerging research has highlighted the potential of natural compounds as antiviral agents effective against resistant viral strains [94]. Some bioactives inhibit viral entry, replication, and assembly, making them promising candidates for therapeutic development against infections such as influenza, herpes simplex virus, and SARS‐CoV‐2 [95]. The complex modes of action reduce the likelihood of resistance development compared to conventional antivirals.

### 3.3 Anticancer and Cytotoxic Activities

Natural bioactive compounds exert anticancer effects through various mechanisms, including the induction of apoptosis, cell cycle arrest, inhibition of angiogenesis, and suppression of metastasis [96]. These compounds modulate key signaling pathways that regulate cell survival and proliferation, including the phosphoinositide 3‐kinase (PI3K)/Akt pathway, the Wnt/β‐catenin signaling, and the tumor suppressor protein p53. For example, genistein, a soy isoflavone, induces cell cycle arrest and apoptosis by modulating the PI3K/Akt and MAPK pathways [97]. Other compounds, such as epigallocatechin gallate (EGCG) from green tea, inhibit angiogenesis and metastasis by downregulating vascular endothelial growth factor (VEGF) [98]. Substantial preclinical studies and several clinical trials have validated the anticancer potential of natural compounds, either alone or as adjuvants, enhancing chemotherapy efficacy and reducing toxicity [99, 100]. Table 2 summarizes the key natural compounds, their molecular targets, and documented anticancer mechanisms, providing an integrated view of their therapeutic actions.

**TABLE 2:** Key natural compounds, their molecular targets, and anticancer mechanisms with references.

Natural compound	Molecular targets/pathways	Anticancer mechanisms	References
2‐Methylpyridine‐1‐ium‐1‐sulfonate	VEGF, MMP‐2, MMP‐9, p21, p27, p53, Bax/Bcl‐2, caspase‐3/‐9	Inhibits angiogenesis, induces apoptosis, causes cell cycle arrest at G0/G1 and S phases	[101]
Flavonoids	NF‐κB, mitochondrial pathways, VEGF	Antiproliferation, cell cycle arrest, apoptosis, inhibits inflammation, angiogenesis, metastasis	[102]
Allyl isothiocyanate (AITC)	Multiple including oxidative stress, inflammation, cell cycle, angiogenesis	Regulates oxidative stress, induces apoptosis, arrests cell cycle, inhibits angiogenesis and metastasis	[103]
Fucoxanthin	VEGF‐A, VEGF‐C, cell cycle regulators	Induces cell cycle arrest, apoptosis; inhibits angiogenesis and migration	[104]
Genistein	Galectin‐3, p21, cell cycle G2/M checkpoint	Induces apoptosis and cell cycle arrest; modulates p21 expression depending on galectin‐3 status	[105]
Thymoquinone	Apoptosis pathways, ROS generation, angiogenesis	Promotes apoptosis, cell cycle arrest, modulates oxidative stress, and metastasis	[106]
Icariside II	Apoptosis, autophagy, cell cycle regulators, angiogenesis	Induces apoptosis, inhibits proliferation, metastasis, angiogenesis; synergizes with chemotherapy	[107]
Eugenol	MAPK/ERK, PI3K/Akt/mTOR, JAK/STAT	Induces apoptosis, cell cycle arrest; anti‐inflammatory; inhibits angiogenesis and metastasis	[108]
Luteolin	PI3K/Akt, NF‐κB, MAPK	Induces apoptosis, cell cycle arrest; inhibits proliferation, angiogenesis, metastasis	[109]
Raddeanin A	PI3K/Akt, Wnt/β‐catenin, NF‐κB, STAT3	Induces apoptosis, cell cycle arrest; inhibits proliferation, invasion, angiogenesis, metastasis	[110]
Evening Primrose Oil	VEGF, cyclin D1, Bax/Bcl‐2, caspase‐3	Induces apoptosis, inhibits angiogenesis, arrests cell cycle; synergistic with tamoxifen	[111]
Mevinolin (statin)	Cell cycle regulators, ROS generation, DNA repair	Induces cell cycle arrest and apoptosis, inhibits proliferation, triggers ROS	[112]
Ellagic acid	TGF‐β1/Smad3 signaling	Induces cell cycle arrest, apoptosis via TGF‐β1/Smad3 pathway	[113]
Propolis (polyphenols/flavonoids)	NF‐κB, MMPs, apoptotic regulators	Induces cell cycle arrest, apoptosis; inhibits invasion, angiogenesis, metastasis; modulates inflammation	[114]
Abietic acid	NF‐κB, PI3K/Akt, AMPK, mitochondrial pathways	Induces apoptosis, cell cycle arrest, inhibits proliferation via multiple signaling pathways	[115]
Galbanic acid	Apoptosis, cell cycle arrest, angiogenesis	Induces apoptosis, inhibits angiogenesis and metastasis; synergizes with chemotherapy	[116]
Plant‐derived bioactives (general)	STAT‐3, PI3K/Akt, Ras/MAPK pathways	Induce apoptosis, cell cycle arrest, inhibit angiogenesis, proliferation, metastasis	[117]

### 3.4 Neuroprotective and Cardiovascular Effects

Natural bioactives contribute substantially to neuroprotection by attenuating oxidative stress and inflammation within the nervous system, as well as modulating neurotransmitter levels [118]. For instance, compounds such as bacosides from *Bacopa monnieri* improve cognitive function by reducing neuronal damage and enhancing synaptic plasticity [119]. Antioxidant properties further protect against neurodegenerative diseases such as Alzheimer's and Parkinson's. In cardiovascular health, natural compounds promote vasodilation, improve lipid metabolism, and exhibit antithrombotic effects [120]. Flavonoids, such as hesperidin and naringenin, improve endothelial function and reduce low‐density lipoprotein (LDL) oxidation, key contributors to atherosclerosis [121]. Moreover, the anti‐inflammatory actions of flavonoids reduce vascular inflammation, thus preventing the progression of cardiovascular diseases.

## 4 Applications of Natural Components

Natural bioactive compounds have permeated diverse sectors due to their wide‐ranging biological activities, natural origin, and consumer preference for safer alternatives. These compounds provide diverse benefits and have been widely adopted in pharmaceuticals, agriculture, food preservation, cosmetics, and nutraceuticals [122]. Furthermore, the integration of natural bioactive compounds into these industries not only reflects their therapeutic and functional potential but also responds to increasing regulatory and environmental demands for sustainability and reduced chemical load. This section provides an in‐depth review of the major applications of natural bioactive compounds, focusing on pharmaceutical and therapeutic uses, as well as roles in the agricultural and food industries, and the incorporation of cosmetics and nutraceuticals.

### 4.1 Pharmaceutical and Therapeutic Applications

The pharmaceutical industry continues to rely heavily on natural bioactive compounds as primary sources for novel drug discovery and development [123]. Historically, many blockbuster drugs have originated from natural products, such as paclitaxel, isolated from the bark of *Taxus brevifolia*, which revolutionized cancer treatment by stabilizing microtubules and inhibiting cell division [124]. Similarly, artemisinin, derived from *Artemisia annua*, has transformed antimalarial therapy globally [44]. Presently, numerous natural compounds and their semisynthetic derivatives are undergoing clinical trials for various diseases, including cancer, neurodegenerative disorders, inflammatory diseases, and infectious diseases [125, 126, 127]. The complex molecular architectures of natural compounds offer unique mechanisms of action, which are often difficult to replicate synthetically, such as multitarget modulation and epigenetic regulation.

However, the pharmaceutical exploitation of natural bioactives faces serious challenges; for example, many compounds demonstrate poor aqueous solubility, which limits their absorption and systemic bioavailability [128]. Additionally, rapid metabolism and clearance of compounds can reduce therapeutic efficacy. Meanwhile, some natural compounds exhibit off‐target toxicity or adverse interactions with conventional drugs [129]. Therefore, addressing these limitations requires innovative formulation strategies, including nanoencapsulation, liposomal delivery, solid dispersions, and conjugation to targeting moieties [130]. These technologies improve solubility, stability, and targeted delivery, enabling controlled release and reduced systemic toxicity [131]. Furthermore, advances in medicinal chemistry facilitate the development of analogs with optimized pharmacokinetic and pharmacodynamic properties [132]. This thorough integration of natural compounds with cutting‐edge drug delivery and design techniques continues to expand the clinical applicability of natural bioactive compounds.

### 4.2 Agricultural and Food Industry Uses

**FIGURE 3:**
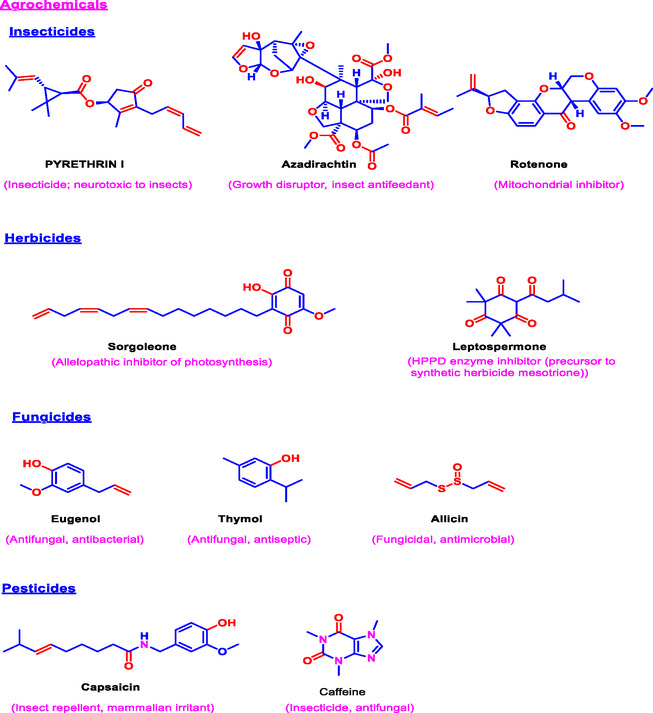
An overview of plant‐derived agrochemicals, including insecticides, herbicides, fungicides, and pesticides.

The agricultural industry has increasingly adopted natural bioactive compounds as sustainable alternatives to synthetic agrochemicals, which pose environmental and health risks [133]. Plant‐derived compounds, such as alkaloids, terpenoids, and phenolics, exhibit strong pesticidal, herbicidal, and fungicidal activities, effectively managing a broad spectrum of pests and pathogens [133]. For example, pyrethrins extracted from *Chrysanthemum* species are widely used as botanical insecticides that degrade rapidly, minimizing environmental persistence [134]. Such compounds contribute to integrated pest management strategies by reducing chemical residues in ecosystems and food products, aligning with global calls for greener agricultural practices (Figure 3).

Beyond pest control, many bioactives function as biostimulants that enhance crop growth, exhibit increased resilience to abiotic stresses, and improve nutrient uptake [135]. These effects are mediated through hormonal modulation and improved photosynthetic efficiency. For instance, seaweed extracts rich in polysaccharides and polyphenols promote plant vigor and yield, especially under drought or salinity stress [136]. These natural enhancers offer a viable route to sustainable intensification of agriculture by improving productivity without adverse environmental impacts.

**FIGURE 4:**
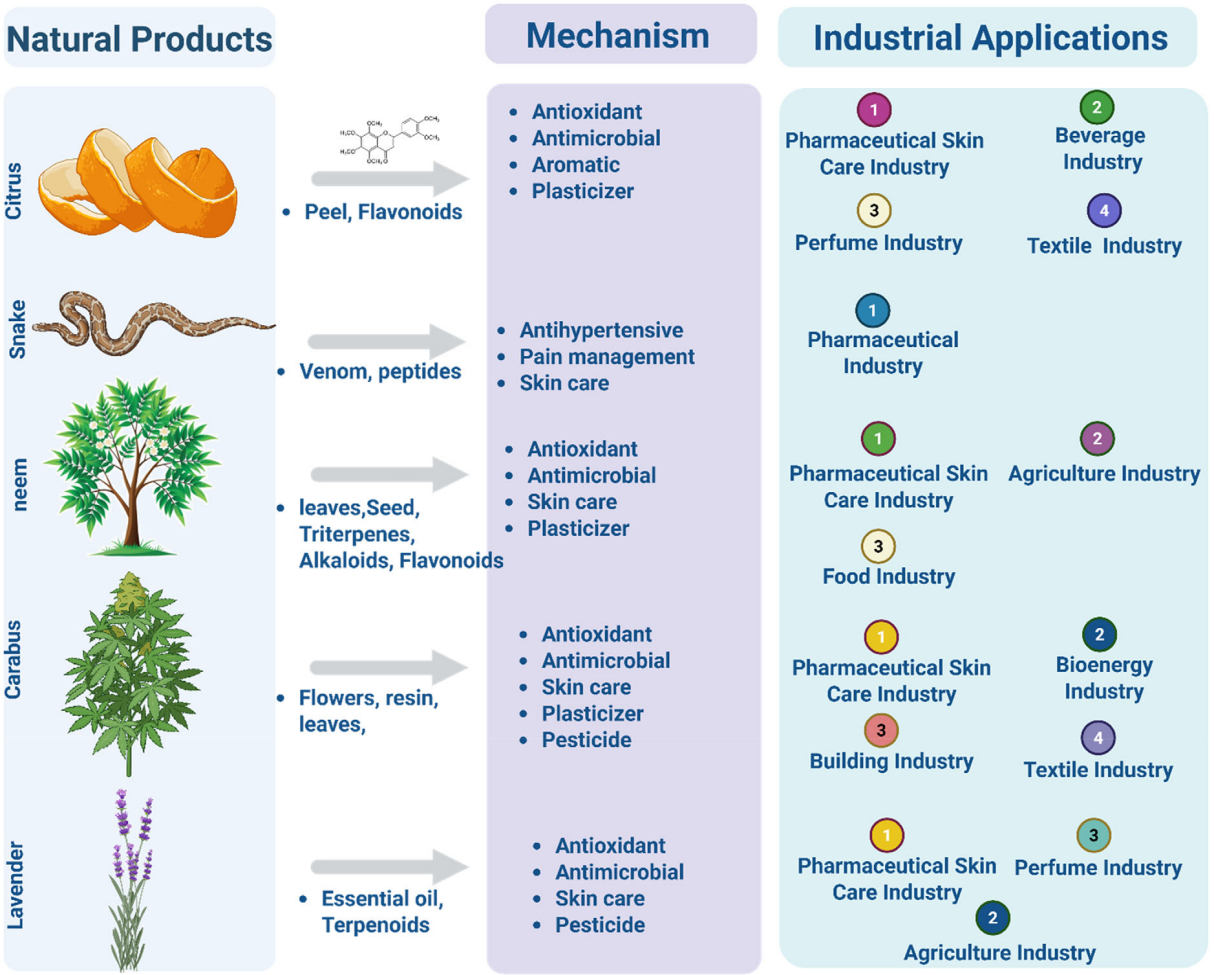
Natural bioactive products, their key constituents, associated biological mechanisms, and diverse industrial applications.

Natural antioxidants and antimicrobials derived from bioactives are widely incorporated in the food industry to extend shelf life and maintain quality [137]. Phenolic compounds inhibit lipid peroxidation and microbial spoilage, thereby preserving the flavor, color, and nutritional value of products ranging from oils to processed meats [138]. Moreover, the incorporation of natural extracts into packaging materials and edible coatings further enhances preservation by providing active barriers to oxidation and microbial growth [139]. This approach reduces reliance on synthetic preservatives, which are often linked to health concerns and consumer skepticism. Figure 4 presents a visual summary of the diverse applications of natural bioactive compounds within agriculture and the food industry, illustrating their roles as natural pesticides, growth promoters, and food preservatives, along with the underlying biochemical pathways and benefits.

### 4.3 Cosmetic and Nutraceutical Applications

The cosmetic industry has embraced natural bioactive compounds due to their multifunctional benefits for skin health, including antioxidant protection, ultraviolet (UV) defense, anti‐inflammatory effects, and the promotion of collagen synthesis [140]. Ingredients such as green tea polyphenols, vitamin C, and carotenoids help mitigate oxidative damage caused by UV radiation and environmental pollutants, thereby reducing photoaging and skin inflammation [141]. The growing consumer demand for “clean” and “natural” products has driven extensive research and formulation efforts to harness these compounds effectively, striking a balance between efficacy, safety, and sensory appeal.

Nutraceuticals and functional foods enriched with natural bioactives can be employed to support health maintenance and chronic disease prevention by providing physiological benefits that extend beyond basic nutrition [142]. Polyphenols, omega‐3 fatty acids, and dietary fibers contribute to cardiovascular health, cognitive function, and metabolic regulation [143]. Meanwhile, growth in this sector is being driven by the rising prevalence of lifestyle‐related diseases and increased health awareness. Products ranging from dietary supplements to fortified beverages incorporate these bioactives, often supported by clinical evidence demonstrating efficacy [144]. Table 3 presents an overview of commercially available cosmetic and nutraceutical products that contain key natural bioactive compounds.

**TABLE 3:** Commercially available cosmetic and nutraceutical products containing natural bioactives, with active ingredients, claimed benefits, and target markets.

Category	Active ingredient(s)	Claimed benefits	Target consumer demographics	References
Cosmetics	Green tea polyphenols (EGCG)	Antioxidant protection, UV defense, anti‐inflammatory, reduces photoaging and skin inflammation	Adults seeking antiaging and skin protection	[145, 146]
	Vitamin C (ascorbic acid)	Promotes collagen synthesis, brightening, antioxidant	Individuals with dull or aging skin	[147, 148]
	Carotenoids (β‐carotene)	Antioxidant	Consumers interested in natural sun protection	[149]
	Hyaluronic acid	Skin hydration, antiwrinkle	Mature skin, dry skin consumers	[150]
	Enzymatically synthesized bioactives	Enhanced skin barrier, anti‐inflammatory, moisturizing	Sensitive skin consumers	[151]
Nutraceuticals	Polyphenols (resveratrol, flavonoids)	Cardiovascular protection, anti‐inflammatory, metabolic regulation	Adults with cardiovascular risk or metabolic concerns	[152, 153]
	Omega‐3 fatty acids (EPA, DHA)	Cognitive function, cardiovascular health, anti‐inflammatory	Aging population, health‐conscious adults	
	Dietary fibers (soluble and insoluble)	Metabolic regulation, gut health, weight management	General health, weight management	
	Probiotics and prebiotics	Gut health, immune modulation	Adults seeking digestive and immune health	
	Plant extracts (e.g., citrus bioactives)	Antioxidant, anti‐inflammatory, metabolic benefits	Consumers seeking natural supplements	[149]

## 5 Advances in Biotechnological Approaches

Recent biotechnological innovations have greatly advanced the sustainable production and enhanced utility of natural bioactive compounds [33]. Traditional extraction from wild or cultivated plants faces challenges, including low yields, seasonal variability, and environmental constraints [154]. Biotechnology offers scalable, controlled, and eco‐friendly alternatives for producing bioactives through plant cell cultures, microbial fermentation, and genetic engineering [155]. These approaches not only ensure consistent quality and supply but also enable the biosynthesis of novel derivatives with improved pharmacological properties. This section reviews key biotechnological production methods, analytical tools for compound discovery, and nanotechnology‐based delivery systems that collectively enhance the application potential of natural bioactives.

### 5.1 Biotechnological Production and Metabolic Engineering

Plant cell cultures and microbial fermentation have emerged as effective platforms for producing high‐value natural compounds under controlled conditions [156]. Plant suspension cultures, hairy root cultures, and organ cultures allow the biosynthesis of secondary metabolites independently of climatic or geographic limitations, reducing harvesting pressures on natural populations [157]. Microbial fermentation, utilizing bacteria, fungi, and yeast that are genetically engineered or naturally capable of producing bioactives, offers advantages such as rapid growth, scalability, and genetic manipulability [158].

Genetic and metabolic engineering strategies further expand the potential of these platforms by modifying biosynthetic pathways to increase yields, reduce by‐products, and generate novel compounds [159]. Techniques such as CRISPR/Cas9 genome editing, pathway overexpression, and synthetic biology enable precise control over enzymatic steps, facilitating the production of complex molecules that would otherwise be difficult to obtain [160]. For example, the metabolic engineering of *Saccharomyces cerevisiae* has enabled the efficient production of artemisinic acid, a precursor to artemisinin, improving access to antimalarial drugs [161].

Nonetheless, despite these successes, bottlenecks remain, including limited understanding of complete biosynthetic pathways, regulatory complexity, and high production costs. Thus, optimizing culture conditions, improving gene expression systems, and integrating multiomics data are required to continue driving progress. These approaches, along with their advantages, limitations, and examples, are summarized in Table 4.

**TABLE 4:** Biotechnological methods for natural bioactive compound production: approaches, advantages, limitations, and representative examples.

Method/approach	Advantages	Limitations	Representative examples	References
Plant cell cultures	Controlled biosynthesis independent of climate/geography. Can produce complex secondary metabolites. Sustainable, reduces wild harvesting	Slow growth rates compared to microbes. Low yield for some metabolites. Complex medium requirements	Taxol production in *Taxus* suspension cultures; Vinblastine in *Catharanthus* cultures	[162, 163]
Hairy root cultures	High genetic stability. High yield of root‐specific metabolites. Faster growth than callus cultures	Limited to root metabolites. Requires transformation with *Agrobacterium rhizogenes*	Production of tropane alkaloids, e.g., hyoscyamine and scopolamine	[164, 165]
Organ cultures	Can mimic whole‐plant metabolic complexity. Can produce metabolites localized to specific organs	Difficult to scale‐up. Labor‐intensive and costly	Ginsenoside production from *Panax* root cultures	[166, 167]
Microbial fermentation	Rapid growth, scalability. Genetic manipulability. Cost‐effective production in bioreactors	Sometimes requires complex pathway engineering for plant metabolites. May produce unwanted by‐products	Artemisinic acid production in engineered *Saccharomyces cerevisiae*	[168]
Metabolic engineering	Increases yield and specificity. Can generate novel compounds. Enables pathway optimization and modular control	Complex regulatory networks. Off‐target effects and metabolic burden. High research and development costs	Engineering *E. coli* and yeast for taxadiene (precursor of Taxol) production	[169]
CRISPR/Cas9 genome editing	Precise gene editing. Multiplex editing capabilities. Enables functional genomics studies	Off‐target mutations possible. Regulatory hurdles for GMO products	Editing *Artemisia annua* genes to enhance artemisinin biosynthesis	[170]
Synthetic biology	Design of artificial biosynthetic pathways. Modular and programable. Potential for novel compound production	High design complexity. Requires extensive metabolic knowledge	Synthetic pathways for opioids and cannabinoids in microbes	[171]
Integration of multiomics data	Holistic pathway analysis. Identifies bottlenecks and regulatory nodes. Guides rational engineering	Large data complexity. Requires bioinformatics expertise	Transcriptomics and metabolomics guiding Taxol production optimization	[172]

### 5.2 Analytical Techniques for Compound Discovery and Characterization

Advanced analytical techniques form the backbone of natural compound discovery and structural characterization. Chromatographic methods, such as HPLC and gas chromatography (GC), facilitate the separation and quantification of complex mixtures [173, 174]. Spectroscopic tools, including MS and NMR spectroscopy, provide detailed molecular structural information essential for identifying and confirming novel compounds [175]. These technologies are integrated through high‐throughput metabolomics to profile entire metabolite pools rapidly, enabling the detection of minor yet biologically relevant compounds [176]. In addition to chromatographic and mass spectrometric methods, spectroscopic and physical techniques are essential for structural elucidation of natural bioactive compounds. Infrared (IR) and ultraviolet–visible (UV–vis) spectroscopy help identify functional groups and conjugated systems, respectively. Optical rotation and circular dichroism (CD) provide insights into stereochemistry, while single‐crystal X‐ray diffraction offers definitive 3D structural information. Together, these methods ensure accurate structure determination and quality control in natural product research. Coupled with bioinformatics and chemometrics, these methods accelerate the screening and elucidation of bioactive molecules from complex biological matrices.

### 5.3 Nanotechnology and Drug Delivery Systems

Nanotechnology has revolutionized the delivery of natural bioactive compounds by overcoming challenges related to solubility, stability, and bioavailability [177]. Nanoparticles, liposomes, micelles, and other nanocarrier systems encapsulate bioactives, protecting them from degradation and facilitating targeted delivery to specific tissues or cells [178]. This encapsulation enhances solubility and absorption, prolongs circulation time, and allows for controlled release, thereby improving therapeutic efficacy and reducing off‐target toxicity [179].

**FIGURE 5:**
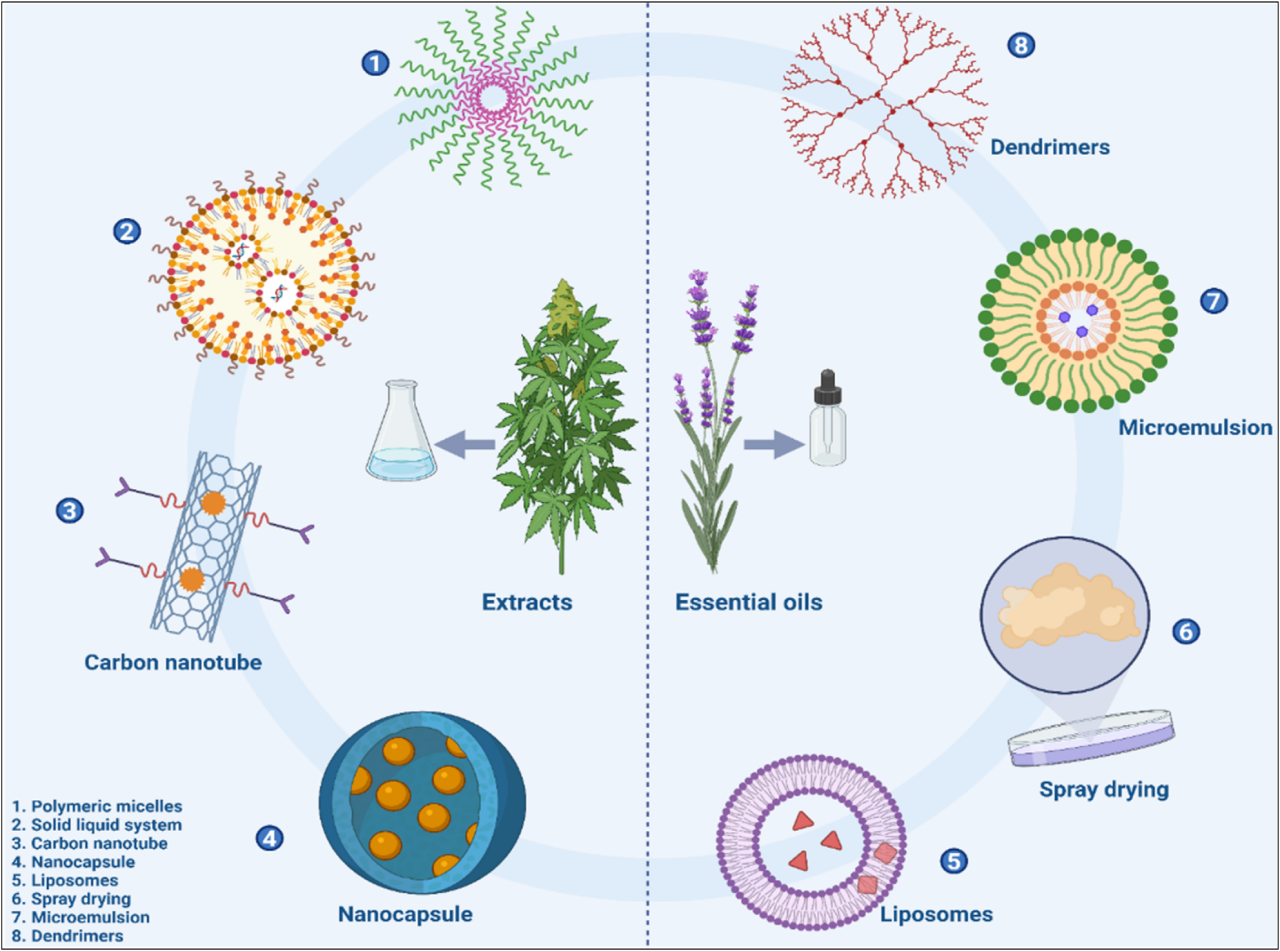
Schematic illustration of nanocarrier systems encapsulating natural bioactive compounds.

For instance, curcumin‐loaded nanoparticles demonstrate enhanced anticancer activity due to improved cellular uptake and retention [180]. Similarly, liposomal formulations of resveratrol have shown increased bioavailability and neuroprotective effects [181]. The versatility of nanocarriers also enables codelivery of multiple agents, synergizing therapeutic actions. A schematic representation of various nanocarrier systems and their functions is illustrated in Figure 5.

## 6 Safety, Toxicity, and Regulatory Aspects

Natural bioactive compounds possess tremendous therapeutic and industrial potential; however, the safe and effective application of these compounds hinges on a thorough evaluation of their pharmacokinetics, toxicity, and adherence to regulatory standards [182]. Moreover, the inherent chemical complexity and variability of these compounds, often extracted from diverse natural sources, introduce challenges in predicting their behavior within biological systems and ensuring consistent product quality [183]. This section provides a comprehensive overview of the pharmacokinetic profiles and toxicological considerations of natural bioactives, followed by a detailed examination of regulatory frameworks and quality control measures that govern the global implementation of these compounds.

### 6.1 Pharmacokinetics and Toxicity Profiles

The pharmacokinetic properties of natural bioactive compounds, including absorption, distribution, metabolism, and excretion (ADME), significantly influence their therapeutic efficacy and safety profiles [184]. Many natural compounds exhibit poor aqueous solubility and low bioavailability, limiting their systemic exposure and pharmacological action [185]. For instance, polyphenols such as quercetin undergo extensive metabolism by intestinal microbiota and hepatic enzymes, leading to a complex mixture of metabolites with varying biological activities and toxicities [186]. Moreover, the presence of efflux transporters, such as P‐glycoprotein, can further restrict intestinal absorption [187]. Such metabolic transformations sometimes result in metabolites that are more active or toxic than the parent compounds, complicating safety assessments.

**FIGURE 6:**
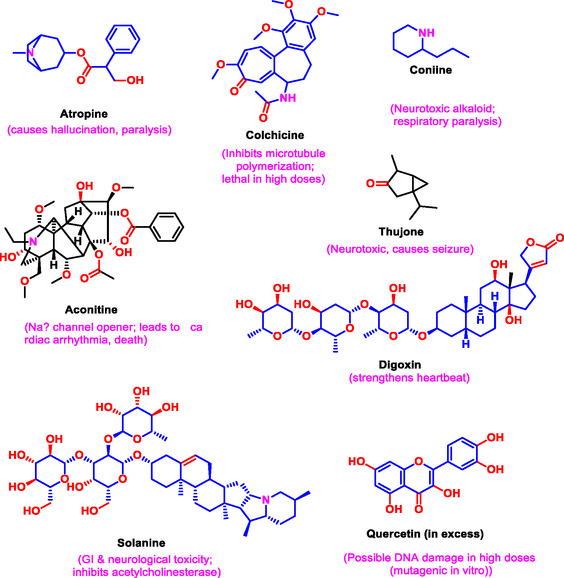
Toxicity of plant‐derived natural products.

Toxicological evaluations encompass acute, sub‐chronic, and chronic studies that elucidate dose‐dependent effects, organ‐specific toxicity, genotoxicity, and carcinogenic potential [188]. However, while many natural products are considered generally safe, notable exceptions exist; for example, pyrrolizidine alkaloids found in some medicinal plants are hepatotoxic and carcinogenic at high exposure levels (Figure 6) [189]. Additionally, interactions between natural compounds and pharmaceuticals may potentiate adverse effects or reduce therapeutic efficacy, a key consideration in polypharmacy contexts [190]. Contaminants such as heavy metals, pesticide residues, and microbial toxins, which are frequently detected in herbal products, also contribute to toxicity risks [191]. Consequently, rigorous toxicological profiling, through in vitro assays, animal studies, and clinical observations, is indispensable for ensuring consumer safety.

### 6.2 Regulatory Challenges and Quality Control

The commercialization of natural bioactive compounds is tightly regulated by agencies such as the U.S. Food and Drug Administration (FDA), the European Medicines Agency (EMA), and similar bodies worldwide [192]. These agencies enforce regulatory frameworks that vary depending on product classification. Products may be categorized as drugs, dietary supplements, or cosmetics, each with specific requirements for safety, efficacy, and quality. One major regulatory challenge is the absence of universally accepted standards for characterizing and quantifying bioactive constituents, leading to significant batch‐to‐batch variability and challenges to the reproducibility of clinical effects [193].

Standardization issues are compounded by potential contamination with heavy metals (e.g., lead, mercury), pesticide residues, microbial pathogens, and adulteration with synthetic pharmaceuticals, all of which pose serious public health hazards [194]. To mitigate these risks, strict adherence to good manufacturing practices (GMPs) and the implementation of quality assurance systems are mandatory [195]. The implementation of analytical techniques, such as chromatographic fingerprinting and DNA barcoding, further supports the authentication and detection of adulterants [37]. Furthermore, regulatory agencies are increasingly demanding robust postmarket surveillance and pharmacovigilance data to monitor adverse events and ensure ongoing product safety [196]. Table 5 presents a summary of the critical safety information, toxicological concerns, and regulatory guidelines pertinent to major natural bioactive compounds, serving as a key reference for scientists, manufacturers, and regulators.

**TABLE 5:** Summary of safety data, toxicological concerns, and regulatory guidelines for widely used natural bioactive compounds.

Aspect	Description	Challenges/concerns	Quality control methods	Regulatory guidelines	References
Regulatory frameworks	Different classifications affect regulatory requirements (drugs, supplements, cosmetics). Regulatory agencies include FDA (USA), EMA (EU), etc	Varying standards across regions; lack of global harmonization complicates commercialization	Clear product classification, labeling, and registration based on regulatory category	FDA Dietary Supplement Health and Education Act (DSHEA); EMA guidelines on herbal medicinal products (EMA/HMPC/214820/2017)	[197]
Standardization of bioactives	Lack of universally accepted standards for bioactive content leads to variability in efficacy and safety	Batch‐to‐batch variability; difficulties in clinical reproducibility	Chromatographic fingerprinting (HPLC, GC–MS), spectroscopic methods, DNA barcoding for plant species authentication	Quality control guidelines in the WHO monographs on selected medicinal plants, ICH Q6A for specifications	[198, 199]
Contamination risks	Heavy metals (lead, mercury), pesticide residues, microbial contamination, and adulteration with synthetic drugs	Public health hazards from contaminants or undeclared substances	ICP–MS for heavy metals; pesticide residue analysis; microbial culture and PCR; chemical screening for adulterants	FDA Guidance for Industry on Botanical Drug Development (2016); EMA Guideline on quality of herbal medicinal products	[200, 201]
Toxicological concerns	Varied toxicity profiles; some bioactives have dose‐dependent adverse effects or interactions with conventional drugs	Acute/chronic toxicity, hepatotoxicity, allergenicity, herb‐drug interactions	Preclinical toxicology studies, postmarket pharmacovigilance, in vitro cytotoxicity assays	Toxicological evaluation frameworks: OECD guidelines; EMA Safety Monitoring of Herbal Medicines; FDA pharmacovigilance requirements	[202, 203]
GMPs	Compliance with GMPs ensures consistency in production quality and safety	Noncompliance leads to poor product quality, contamination, and safety risks	GMP certification, quality assurance protocols, regular audits	WHO GMP guidelines for herbal medicines; FDA GMP regulations (21 CFR Part 111) for dietary supplements	[204, 205]
Authentication and adulteration detection	DNA barcoding and chromatographical fingerprinting is used detect species substitution and adulteration with synthetic compounds	Fraudulent substitution; adulteration affecting safety and efficacy	DNA barcoding, LC–MS/MS metabolite profiling, chemical marker analysis	Research studies: Newmaster et al. (2013) on DNA barcoding in herbal supplements	[206]
Postmarket surveillance and pharmacovigilance	Continuous monitoring of adverse effects once products are marketed	Under‐reporting of adverse events; lack of comprehensive databases	Adverse event reporting systems, safety monitoring databases, product recalls	FDA MedWatch; EMA EudraVigilance; WHO VigiBase for herbal medicines	[207]
Dose and safety ranges	Recommended safe dosage ranges are essential to avoid toxicity and ensure efficacy	Variability in traditional usage versus scientific dosage guidelines	Clinical trials, toxicology studies, literature meta‐analyses	Clinical trial guidelines: FDA Botanical Drug Development Guidance (2016); EMA Clinical Trials Regulation	[208, 209]
Documentation and labeling requirements	Accurate ingredient listing, health claims, warnings, and expiration information	Misleading labels; undeclared allergens or substances	Regulatory audits, certification schemes (e.g., NSF International, USP)	FDA labeling regulations for supplements; EMA labeling requirements for herbal products	[210]

### 6.3 Standardization and Authentication

The authenticity and standardization of natural bioactive products are essential to guarantee their efficacy, safety, and consumer trust [211]. Botanical authentication techniques, such as DNA barcoding, chromatographic fingerprinting (HPLC and TLC), and spectroscopic profiling (NMR and FTIR), enable the precise identification of plant species and the detection of adulteration or substitution with inferior or harmful materials [37]. These methods provide objective, reproducible, and sensitive tools to safeguard the integrity of raw materials and finished products throughout the supply chain.

Moreover, good agricultural practices (GAPs) and GMPs ensure quality from cultivation to processing [212]. GAPs encompass the selection of plant varieties, soil and water management, pest control, and harvesting conditions, all of which influence the concentration and consistency of bioactive constituents [213]. GMPs also govern production processes, including extraction, formulation, packaging, and storage, to prevent contamination and ensure product uniformity [214]. Collectively, these measures underpin the standardization processes imperative for natural bioactives, fostering regulatory compliance and consumer confidence.

## 7 Current Challenges and Future Perspectives

The field of natural bioactive compounds faces numerous challenges arising from the inherent complexity and variability of natural products, which affect reproducibility and translational success in research and application [215]. Natural extracts are complex mixtures containing a vast array of chemically diverse compounds that often interact synergistically or antagonistically, complicating the elucidation of mechanisms of action and standardization efforts [215]. Indeed, variability in chemical composition, due to genetic diversity, environmental factors such as soil quality and climate, harvesting time, and processing methods, further exacerbates the difficulty of producing consistent and reproducible outcomes [216]. These factors contribute to conflicting data in experimental research, thereby limiting the reliability of clinical studies and hindering the regulatory approval and commercialization of new treatments.

Thus, to address these complexities, recent technological advances have introduced artificial intelligence (AI) and ML as powerful tools [217]. AI‐driven predictive models facilitate the identification and prioritization of bioactive compounds by analyzing large chemical and biological datasets, uncovering hidden patterns and relationships that traditional methods may overlook [12]. ML algorithms facilitate virtual screening, activity prediction, and toxicity assessment, substantially accelerating the discovery pipeline and reducing experimental costs [218]. Coupled with natural language processing and data mining, AI platforms also extract valuable insights from the vast and growing body of scientific literature and chemical databases [12]. These advances foster a more systematic and data‐driven approach to natural product research, enhancing reproducibility and translational potential.

The integration of multiomics data provides unprecedented mechanistic insights into the biosynthesis, regulation, and biological effects of natural compounds [219]. These data include genomics, transcriptomics, proteomics, metabolomics, and epigenomics. Thus, by correlating gene expression profiles with metabolite accumulation and protein function, scientists can gain a deeper understanding of the complex biological networks and pathways modulated by bioactives [220]. This integrative approach enables the identification of biomarkers for efficacy and toxicity, supports metabolic engineering efforts, and aids in personalized medicine strategies. Nonetheless, despite these benefits, challenges remain in data integration, standardization, and interpretation, requiring sophisticated computational tools and interdisciplinary expertise.

Sustainability and conservation of natural resources present major concerns as the global demand for bioactive compounds increases [221]. Overharvesting of medicinal plants and unsustainable collection practices threaten biodiversity and ecosystem stability, risking the loss of valuable genetic resources [222]. Climate change further impacts plant distribution and secondary metabolite profiles, potentially altering the availability and efficacy of compounds. Addressing these issues requires the adoption of sustainable harvesting protocols, cultivation of medicinal plants, and the development of alternative production methods, such as microbial biosynthesis and synthetic biology [223]. Policymaking, community engagement, and global cooperation are essential to balance utilization with conservation.

Overall, interdisciplinary collaboration and innovation are required to overcome the challenges posed by the complexity of natural products, sustainability, and data integration. Hence, chemists, biologists, data scientists, agronomists, and regulatory experts must collaborate to develop robust methodologies, innovative technologies, and comprehensive frameworks that advance the research and application of natural bioactive compounds. Such collective efforts will unlock the full potential of the chemical diversity present in nature for health, agriculture, and industry.

## 8 Conclusions

Natural bioactive compounds are pivotal in biomedical research and industrial applications due to their diverse chemical structures and varied biological activities. They show promise in treating various health issues, including inflammation, infections, cancer, and neurodegenerative diseases, and are used in agriculture, food preservation, and cosmetics. Advances in biotechnology, including plant cell culture and microbial fermentation, have enhanced scalability and sustainability.

Meanwhile, challenges persist in standardizing natural extracts, addressing issues such as poor solubility, low bioavailability, and toxicity, as well as concerns about overharvesting. Thus, a multidisciplinary approach combining biotechnology, computational biology, and sustainable resource management is key to overcoming these obstacles. Emerging innovations in synthetic biology, including pathway engineering and genome editing, offer powerful strategies for enhancing the yield and diversity of bioactive compounds. Multiomics technologies, which integrate genomics, transcriptomics, proteomics, and metabolomics, enable a systems‐level understanding of biosynthetic pathways and help unlock previously inaccessible natural products. In parallel, AI‐driven predictive modeling and data integration are transforming natural product discovery by enabling faster identification of active compounds and their molecular targets. Furthermore, nanotechnology continues to improve delivery systems, enhancing therapeutic efficacy and reducing toxicity.

In summary, natural bioactive compounds offer sustainable solutions to health and environmental challenges. Realizing their full potential requires a forward‐thinking framework that integrates synthetic biology, AI, and multiomics approaches with ecological and ethical considerations. This convergence of disciplines offers a transformative path toward unlocking the full therapeutic and industrial potential of natural bioactive compounds.

## Author Contributions

Conceptualization, writing – original draft preparation, resources, software, validation, visualization, S.A., A.A.K.K., M.S.A., and W.Z.; writing – review and editing, validation, supervision, A.A., and W.Z. All authors have read and agreed to the published version of the manuscript.

## Conflicts of Interest

The authors declare no conflicts of interest.

## Data Availability

The data that support the findings of this study are available from the corresponding author upon reasonable request.
